# “An unusual cause of small bowel obstruction: Gallstone ileus, a rare presentation of chronic gallstone disease”

**DOI:** 10.1016/j.ijscr.2025.111894

**Published:** 2025-09-04

**Authors:** Agrawal Srikant, Paudel Pratiksha, Khatiwada Bidur, Manandhar Kishor

**Affiliations:** a National Academy of Medical Sciences, NAMS, Bir Hospital, Department of General Surgery, Kathmandu, Nepal; b Bharatpur Hospital, Kathmandu, Nepal; c Nepalese Army Institute of Health Sciences, Kathmandu, Nepal

**Keywords:** Gallstone, Ileus, Small bowel obstruction, Cholecysto-duodenal fistula, Case report

## Abstract

**Introduction and importance:**

Gallstone ileus is a rare cause of mechanical small bowel obstruction, accounting for 1–4 % of cases and is most common in elderly females. It arises from the passage of a large gallstone through a cholecysto-enteric fistula into the intestinal lumen, where it lodges—typically in the terminal ileum—causing obstruction.

**Presentation of case:**

A 52-year-old male presented with four days of abdominal pain, vomiting, distension, and constipation. He had a 22-year history of gallstones but no prior biliary symptoms. Imaging revealed small bowel obstruction with a cholecysto-duodenal fistula and a 3.5 cm gallstone impacted in the ileum. He underwent successful open enterolithotomy with an uneventful recovery.

**Clinical discussion:**

Gallstone ileus commonly affects elderly females due to higher prevalence of gallstones and chronic cholecystitis. Risk factors for gallstones include obesity, diabetes, and smoking, none of which were present in this patient. Delayed presentation may be attributed to low awareness, limited access to care, and absence of prior symptoms. CT abdomen is the gold standard for diagnosing gallstone ileus, surpassing X-ray and ultrasound, revealing Rigler's triad—pneumobilia, ectopic stone, and obstruction. Surgical options include enterolithotomy alone or combined with cholecystectomy and fistula repair. In stable patients, enterolithotomy alone is preferred due to lower morbidity.

**Conclusion:**

Gallstone ileus should be suspected in bowel obstruction even in atypical populations. Prompt diagnosis and minimally invasive surgical management can ensure good outcomes.

## Highlights

• Gallstone ileus must be suspected in males too, as it can rarely present in middle-aged patients with bowel obstruction signs.
• CT abdomen is the gold standard for diagnosing gallstone ileus, surpassing X-ray and ultrasound in detecting Rigler’s triad.
• Enterolithotomy alone offers lower risk and effective treatment for gallstone ileus in stable patients without active biliary symptoms.
• Delayed diagnosis occurs in atypical cases due to absent biliary history, low awareness, and limited access to early medical care.
• Fistulas may heal spontaneously post-surgery, making single-stage enterolithotomy sufficient in selected uncomplicated cases.

## 1 Introduction

Biliary pathology is a common finding in the general population. Biliary stones represent most of such pathologies mostly occurring in the gall bladder. Such stones include pigment stones, cholesterol stones, or mixed stones. The common presenting symptoms of biliary stones include episodic pain in the right upper quadrant of the abdomen associated with fatty meals. A diverse complication could arise resulting in inflammation of the gallbladder or biliary tract. In some cases, such stones may migrate to the biliary tract or the duodenum. The proximity of the gallbladder to the duodenum can result in a fistula between the organs. This may depend on the size of the stone and the longstanding pathology. As a result, the stones may migrate to the small intestine and lodge in the terminal ileum where the lumen is narrow [1,2]. This can result in abdominal pain, abdominal distension, and vomiting, the cardinal signs of small bowel obstruction. Gallstone ileus commonly occurs in elderly females as imperative from the fact that the epidemiology of biliary pathologies is more common in females [1]. Here we present a case with similar pathology in a middle-aged male presenting with symptoms and signs of small bowel obstruction from a gallstone. This case has been reported in line with the revised SCARE guidelines, 2025 [3].

## 2 Case presentation

A 52-year-old male presented to our emergency department with a sudden onset of abdominal pain, vomiting, and abdominal distension for 4 days. Abdominal pain was periumbilical, mild to moderate in intensity, colicky, radiating all over the abdomen, and exacerbated by feeding and was associated with multiple episodes of bilious vomiting and nausea. He also complained of gradually progressing abdominal distension and no passage of stool and flatus for 2 days. He mentioned having gallstone disease for the last 22 years. He had no history of abdominal pain in the past, yellowish discoloration of the body, melena, per rectal bleeding, hematemesis, dysphagia, recent weight loss, or abnormal skin tags. He did not consume alcohol or use tobacco products. He had not previously received blood products and been operated on. Gallstones were first detected incidentally during an abdominal ultrasound performed 22 years ago in adolescence for non-specific abdominal discomfort, though the patient remained asymptomatic and did not pursue further evaluation or treatment.

**Fig. 1:**
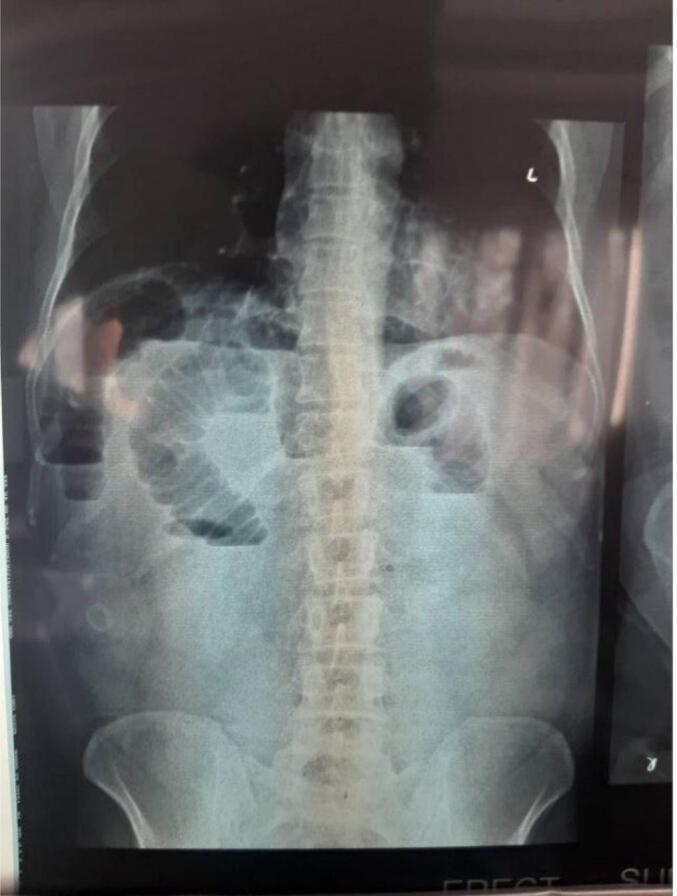
Plain X-ray Abdomen in Erect view showing air-fluid levels and a radio-opaque shadow in the right iliac fossa.

**Fig. 2:**
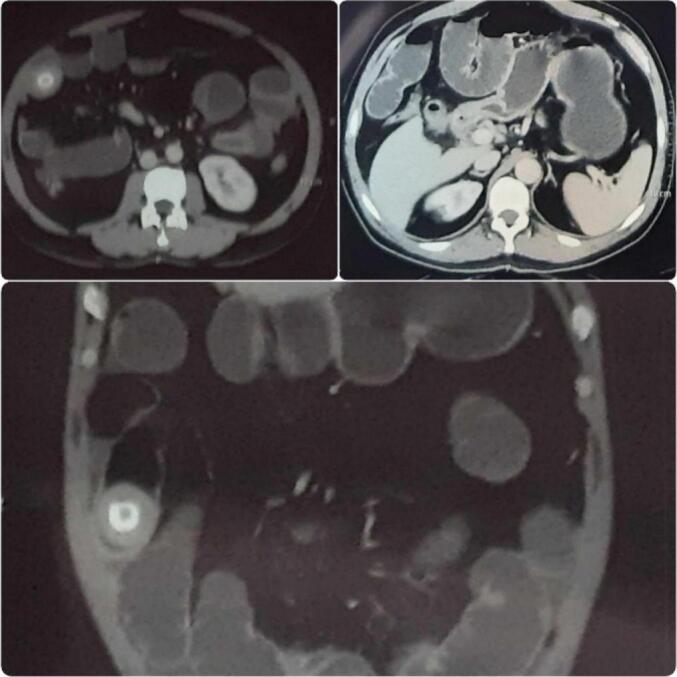
CECT of abdomen and pelvis showing cholecysto-duodenal fistula with gall stone impaction in mid portion of ileum with features suggestive of small bowel obstruction.

On clinical examination, he was tachycardic and had raised blood pressure (160/100 mmHg on his right arm in a recumbent position). He had abdominal distension and bowel sounds were sluggish. The examination findings of other systems were unremarkable. With a provisional diagnosis of partial small intestinal obstruction, he was admitted and evaluated for the possible cause. Baseline investigations including total count, differential count, hemoglobin level, and renal function tests were normal. Ultrasonography of the abdomen revealed a normal scan. X-ray of the abdomen supine film showed multiple dilated bowel loops arranged in the center of the abdomen with valvulae conniventes and in erect film, multiple air-fluid levels were visible along with a radio-opaque shadow with radiolucent centre in the right iliac fossa region (Fig. 1). A contrast-enhanced computed tomography (CECT) of the abdomen and pelvis revealed cholecysto-duodenal fistula with gallstone impaction in the mid portion of the ileum with features suggestive of small bowel obstruction along with mild ascites (Fig. 2).

**Fig. 3:**
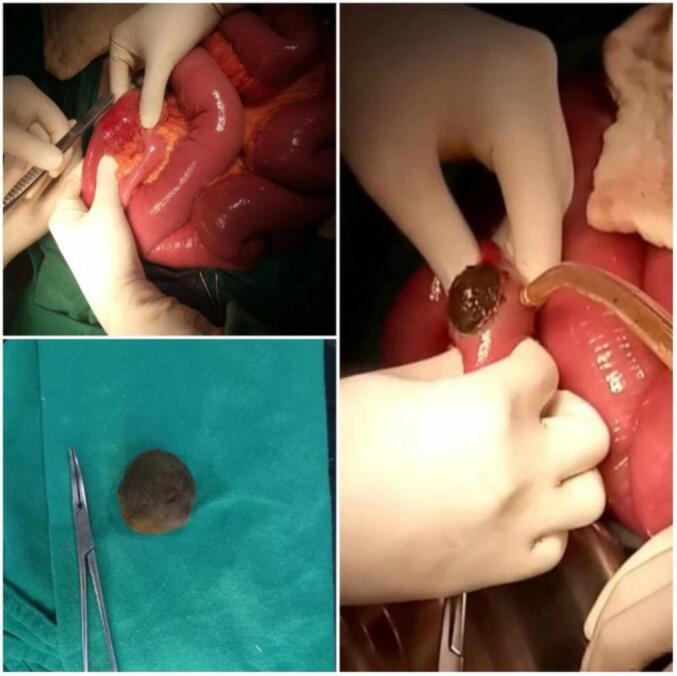
Intraoperative photograph showing a 3.5 cm black stone causing intestinal obstruction.

He was subsequently planned for an emergent surgical intervention. The patient was resuscitated, and an exploratory laparotomy was performed which revealed approximately 100 ml of serous collection in the peritoneal cavity. About 60 cm proximal to the ileocecal junction, obstruction was noted with the proximal loops being dilated and oedematous while the distal ileal loops were collapsed. A simple enterotomy was performed at the level of the obstruction that revealed a blackish-coloured hard stone approximately 3.5 cm in diameter causing a luminal obstruction (Fig. 3). The enterotomy was closed horizontally in two layers. Peritoneal lavage was done. His postoperative hospital stay was not associated with complications and was discharged on the 4th postoperative day. Follow-up evaluations were conducted at 1, 2, and 6 months postoperatively. The patient remained asymptomatic with no signs of biliary complications or recurrent obstruction. Clinical examination and routine laboratory tests were unremarkable, and no further imaging was deemed necessary.

## 3 Discussion

Gallstone ileus accounts for 1–4 % of total admissions for mechanical small bowel obstruction [2]. Among those with gallstone ileus, about 85 % are elderly females with a median age of 70 years [1]. Most of such cases have associated co-morbidities and require a strong suspicion for diagnosis. Only 25–72 % of the cases have a preceding history of biliary pathology. The risk factors for gallstone disease include dietary factors, smoking, obesity, and diabetes [4]. In our case, the patient is a middle-aged male with a 22-year-long history of biliary stones and no associated risk factors or comorbidities. The absence of abdominal pain, lack of knowledge regarding the complications, financial constraints, and difficult access to health care facilities due to geographical location could have played a role in delayed management of his gallstone disease leading to gallstone ileus.

A longstanding history of gallstones could result in repeated attacks of cholecystitis that result in gallbladder wall inflammation and ischemia. This leads to the formation of a fistulous tract between the gallbladder and nearby organs- the duodenum, stomach, bile duct, or colon. Cholecysto-duodenal fistula is present in most such cases. This results in the migration of gallstones to the small bowel, and symptoms occur owing to the size of the stone and the intestinal luminal diameter. Small stones could pass through stool and have no symptoms [4,5]. Larger stones of diameter ≥ 2 cm could cause intermittent obstruction due to tumbling motility or result in complete small bowel obstruction [1]. In our case, the stone size was 3.5 cm.

Abdominal pain is the most common complaint followed by vomiting, abdominal distention, and constipation [4,6]. Our patient had all cardinal signs of small bowel obstruction. The mean time from the development of symptoms to hospital admission is 5.5 days [6]. This is in accordance with the 4-day delay seen in our patient. Plain abdominal radiograph showing pneumobilia, small bowel obstruction, and ectopic gallstone in the intestine, known as Rigler's triad, is present in only 20–35 % of the cases [1]. Ultrasonography could also detect these changes along with the fistulous tract. However, a computed tomography scan of the abdomen and pelvis is considered the best imaging modality in gallstone ileus [4]. In this case, plain radiographs and ultrasonography were inconclusive and a CECT scan made a definitive preoperative diagnosis.

There has been much debate about the best surgical management approach for gallstone ileus. Enterolithotomy alone with stone extraction, enterolithotomy and cholecystectomy with fistula repair either as a single or two-stage procedure, small bowel resection and primary anastomosis along with or without cholecystectomy and fistula repair, shock wave lithotripsy or laparoscopic approach have been mentioned in various studies [1,2,5]. However, enterolithotomy with stone extraction alone is associated with lower mortality rates compared to other approaches [1,7]. We also performed an exploratory laparotomy with stone extraction alone by a longitudinal incision in the antimesenteric border of the ileum. His postoperative course was unremarkable, and the patient did not report any biliary symptoms after a 2-month follow-up. The absence of comorbidities and spontaneous resolution of the fistulous tract could be an important factor for this finding. A Systematic Review and Meta-Analysis of the Management of Gallstone Ileus” showed a significant reduction in mortality with combined entero-lithotomy and cholecystectomy (OR: 2.39 [95 % CI: 1.87, 3.04], I^2^ = 33 %), compared to entero-lithotomy alone (OR: 3.09 [95 % CI: 1.36, 7.02], I^2^ = 69 %) [8].

## 4 Conclusion

Gallstone ileus needs a high index of suspicion for diagnosis in middle-aged males without risk factors for biliary pathology and presenting with symptoms and signs of small intestinal obstruction. Enterolithotomy with stone extraction alone results in favourable postoperative outcomes if the patient has no associated co-morbidities.

## Author contribution

Constructing a hypothesis for the manuscript- Srikant Agrawal, Kishor Manandhar

Planning methodology to reach the conclusion: Srikant Agrawal

Organizing and supervising the course of the article and taking responsibility: Srikant Agrawal, Kishor Manandhar

Patient follow-up and reporting – Pratiksha Paudel, Bidur Khatiwada, Kishor Manandhar

Logical interpretation and presentation of the results- Srikant Agrawal, Pratiksha Paudel, Bidur Khatiwada, Kishor Manandhar

Construction of the whole or body of the manuscript- Srikant Agrawal, Pratiksha Paudel, Bidur Khatiwada, Kishor Manandhar

Reviewing the article before submission not only for spelling and grammar but also for its intellectual content- Srikant Agrawal, Pratiksha Paudel, Bidur Khatiwada, Kishor Manandhar

## Consent

Written informed consent was taken from the patient who participated in this study for publication of this case report and accompanying images.

## Ethical approval

The IRB at our institution has waived ethical approval for case reports.

## Guarantor

The guarantor is Srikant Agrawal.

## Sources of funding

There are no sources of funding for this case study to declare.

## Declaration of competing interest

The authors have no conflict of interest to declare.
